# Supplementary material for: Machine Learning Accelerates Crystallization for Structure Determination
Source: Angew Chem Int Ed Engl. 2026 May 4;65(25):e1218503. doi: 10.1002/anie.1218503 (PMC13266958; doi:10.1002/anie.1218503)

checkCIF/PLATON report

Structure factors have been supplied for datablock(s) lcz098

THIS REPORT IS FOR GUIDANCE ONLY. IF USED AS PART OF A REVIEW PROCEDURE FOR PUBLICATION, IT SHOULD NOT REPLACE THE EXPERTISE OF AN EXPERIENCED CRYSTALLOGRAPHIC REFEREE.

No syntax errors found. CIF dictionary Interpreting this report

Datablock: lcz098

```
Bond precision:   C-C = 0.0212 A          Wavelength=1.54184

Cell:              a=6.5206(2)      b=23.6115(5)      c=21.1404(5)
                   alpha=90         beta=90           gamma=90
Temperature:       100 K
```

	Calculated	Reported
Volume	3254.80(14)	3254.80(14)
Space group	C m c m	C m c m
Hall group	-C 2c 2	-C 2c 2
Moiety formula	C15 H3 Ag3 F18 N6, 4(C5.75 H3.75)	C15 H3 Ag3 F18 N6, 4(C5.75 H3.75)
Sum formula	C38 H18 Ag3 F18 N6	C38 H18 Ag3 F18 N6
Mr	1224.19	1224.19
Dx,g cm-3	2.498	2.498
Z	4	4
Mu (mm-1)	15.734	15.734
F000	2364.0	2364.0
F000’	2374.67	
h,k,lmax	8,29,26	7,29,26
Nref	1953	1842
Tmin,Tmax	0.093,0.110	0.043,1.000
Tmin’	0.009	

```
Correction method= # Reported T Limits: Tmin=0.043 Tmax=1.000
AbsCorr = MULTI-SCAN
```

Data completeness= 0.943 Theta(max)= 78.010

R(reflections)= 0.0914(1603) wR2(reflections)= 0.2725(1842)

S = 1.257 Npar= 199

The following ALERTS were generated. Each ALERT has the format
test-name_ALERT_alert-type_alert-level.
Click on the hyperlinks for more details of the test.

Alert level B

```
PLAT342_ALERT_3_B Low Bond Precision on  C-C Bonds ..............    0.02117 Ang.
```

Author Response: Disordered structure.

Alert level C

```
PLAT084_ALERT_3_C High wR2 Value (i.e. > 0.25) ..................       0.27 Report
PLAT088_ALERT_3_C Poor Data / Parameter Ratio ...................       9.26 Note
PLAT213_ALERT_2_C Atom F00D            has ADP max/min Ratio .....        3.2 prolat
PLAT218_ALERT_3_C Constrained U(i,j) Components(s) for C3                   2 Check
PLAT234_ALERT_4_C Large Hirshfeld Difference N008     --C00C     .       0.16 Ang.
PLAT241_ALERT_2_C High   'MainMol' Ueq as Compared to Neighbors of       C00C Check
PLAT242_ALERT_2_C Low    'MainMol' Ueq as Compared to Neighbors of       C00B Check
PLAT250_ALERT_2_C Large U3/U1 Ratio for <U(i,j)> Tensor(Resd    1)        2.1 Note
PLAT260_ALERT_2_C Large Average Ueq of Residue Including        C1      0.103 Check
PLAT334_ALERT_2_C Small <C-C> Benzene Dist.   C1       -C8       .       1.35 Ang.
PLAT334_ALERT_2_C Small <C-C> Benzene Dist.   C1       -C8       .       1.35 Ang.
PLAT906_ALERT_3_C Large K Value in the Analysis of Variance ......      2.621 Check
PLAT911_ALERT_3_C Missing FCF Refl Between Thmin & STh/L=    0.600         33 Report
                2  0  0,   4  0  0,   1  1  0,   1  3  0,   2  4  0,   1 11  0,
                2 12  0,   1  1  1,   7  3  1,   2  4  1,   1  5  1,   7  5  1,
                2 12  1,   0 22  1,   1  1  2,   0  2  2,   2  2  2,   7  3  2,
                2  4  2,   1  7  2,   6  6  8,   1 15  8,   6  4  9,   6  6  9,
                6  8  9,   7  3 11,   6  0 12,   6  2 15,   6  4 15,   6  0 16,
                   (    3 More Missing: see the .ckf listing file)
PLAT971_ALERT_2_C Check Calcd Resid. Dens.  1.00Ang From C5             2.30 eA-3
PLAT971_ALERT_2_C Check Calcd Resid. Dens.  1.02Ang From C6             1.99 eA-3
PLAT971_ALERT_2_C Check Calcd Resid. Dens.  0.22Ang From C00C           1.75 eA-3
PLAT971_ALERT_2_C Check Calcd Resid. Dens.  0.97Ang From C3             1.52 eA-3
PLAT972_ALERT_2_C Check Calcd Resid. Dens.  0.12Ang From C3            -2.04 eA-3
PLAT972_ALERT_2_C Check Calcd Resid. Dens.  0.13Ang From C2            -1.69 eA-3
PLAT972_ALERT_2_C Check Calcd Resid. Dens.  0.13Ang From C2            -1.69 eA-3
PLAT972_ALERT_2_C Check Calcd Resid. Dens.  0.10Ang From C4            -1.67 eA-3
PLAT972_ALERT_2_C Check Calcd Resid. Dens.  0.45Ang From C8            -1.61 eA-3
PLAT972_ALERT_2_C Check Calcd Resid. Dens.  0.20Ang From C5            -1.59 eA-3
PLAT972_ALERT_2_C Check Calcd Resid. Dens.  0.75Ang From Ag01          -1.57 eA-3
PLAT972_ALERT_2_C Check Calcd Resid. Dens.  1.68Ang From Ag02          -1.57 eA-3
PLAT972_ALERT_2_C Check Calcd Resid. Dens.  0.27Ang From C1            -1.56 eA-3
PLAT973_ALERT_2_C Check Calcd Positive Resid. Density on      Ag01      1.06 eA-3
PLAT976_ALERT_2_C Check Calcd Resid. Dens.  0.64Ang From N2      .     -0.96 eA-3
```

```
PLAT977_ALERT_2_C Check Negative Difference Density on H12       .      -0.41 eA-3
PLAT977_ALERT_2_C Check Negative Difference Density on H7        .      -0.42 eA-3
```

Alert level G

```
PLAT002_ALERT_2_G Number of Distance or Angle Restraints on AtSite          5 Note
PLAT003_ALERT_2_G Number of Uiso or U(i,j)  Restrained non-H-Atoms         18 Report
PLAT072_ALERT_2_G SHELXL First  Parameter in WGHT  Unusually Large       0.20 Report
PLAT171_ALERT_4_G The CIF-Embedded .res File Contains EADP Records          1 Report
PLAT172_ALERT_4_G The CIF-Embedded .res File Contains DFIX Records          1 Report
PLAT174_ALERT_4_G The CIF-Embedded .res File Contains FLAT Records          2 Report
PLAT176_ALERT_4_G The CIF-Embedded .res File Contains SADI Records          4 Report
PLAT177_ALERT_4_G The CIF-Embedded .res File Contains DELU Records          3 Report
PLAT178_ALERT_4_G The CIF-Embedded .res File Contains SIMU Records          4 Report
PLAT186_ALERT_4_G The CIF-Embedded .res File Contains ISOR Records          4 Report
PLAT188_ALERT_3_G A Non-default SIMU Restraint Value has been used     0.0200 Report
PLAT188_ALERT_3_G A Non-default SIMU Restraint Value has been used     0.0100 Report
PLAT188_ALERT_3_G A Non-default SIMU Restraint Value has been used     0.0200 Report
PLAT191_ALERT_3_G A Non-default SADI Restraint Value has been used     0.0100 Report
PLAT191_ALERT_3_G A Non-default SADI Restraint Value has been used     0.0100 Report
PLAT191_ALERT_3_G A Non-default SADI Restraint Value has been used     0.0100 Report
PLAT191_ALERT_3_G A Non-default SADI Restraint Value has been used     0.0100 Report
PLAT192_ALERT_3_G A Non-default DELU Restraint Value for SecondPar     0.0200 Report
PLAT242_ALERT_2_G Low    'MainMol' Ueq as Compared to Neighbors of       C00K Check
PLAT242_ALERT_2_G Low    'MainMol' Ueq as Compared to Neighbors of       C00L Check
PLAT242_ALERT_2_G Low    'MainMol' Ueq as Compared to Neighbors of       C00P Check
PLAT299_ALERT_4_G Atom Site Occupancy Constrained at ............         0.5 Check
              C1      C2      C3      C4      C5      C6      C8      C9
              C10     C11     C12     H2      H3      H5      H6      H9
              H11     H12
PLAT300_ALERT_4_G Atom Site Occupancy of C7         Constrained at       0.25 Check
PLAT300_ALERT_4_G Atom Site Occupancy of H7         Constrained at       0.25 Check
PLAT302_ALERT_4_G Anion/Solvent/Minor-Residue Disorder (Resd    2)       100% Note
PLAT304_ALERT_4_G Non-Integer Number of Atoms in ..... (Resd    2)       9.50 Check
PLAT432_ALERT_2_G Short Inter X...Y Contact  N008      ..C5       .       2.95 Ang.
                                                     -x,y,z  =     12_555 Check
PLAT432_ALERT_2_G Short Inter X...Y Contact  N008      ..C5       .       2.95 Ang.
                                                    1+x,y,z  =      1_655 Check
PLAT720_ALERT_4_G Number of Unusual/Non-Standard Labels ..........         19 Note
              Ag01    Ag02    F003    F004    F005    N008    F009    F00A
              C00B    H00B    C00C    F00D    C00E    C00F    C00I    H00I
              C00K    C00L    C00P
PLAT789_ALERT_4_G Atoms with Negative _atom_site_disorder_group  #         20 Check
PLAT822_ALERT_4_G CIF-embedded .res Contains Negative PART Numbers          1 Check
PLAT860_ALERT_3_G Number of Least-Squares Restraints ............         301 Note
PLAT912_ALERT_4_G Missing # of FCF Reflections Above STh/L=  0.600         56 Note
PLAT913_ALERT_3_G Missing # of Very Strong Reflections in FCF ....          1 Note
                2  0  0,
PLAT933_ALERT_2_G Number of HKL-OMIT Records in Embedded .res File         30 Note
                0  2  2,   0 22  1,   1  1  0,   1  1  1,   1  1  2,   1  3  0,
                1  5  1,   1  7  2,   1  9 19,   1 11  0,   1 15  8,   2  0 20,
                2  2  2,   2  4  0,   2  4  1,   2  4  2,   2 12  0,   2 12  1,
                4  0  0,   5  1 17,   6  0 16,   6  2 15,   6  4  9,   6  4 15,
                6  6  8,   6  6  9,   6  8  9,   6 10 16,   7  3  2,   7  3 11,
PLAT941_ALERT_3_G Average HKL Measurement Multiplicity ..........         2.9 Low
PLAT969_ALERT_5_G The 'Henn et al.' R-Factor-gap value ..........       6.702 Note
              Predicted wR2: Based on SigI**2  4.07 or SHELX Weight 21.69
```

```
PLAT978_ALERT_2_G Number C-C Bonds with Positive Residual Density.          2 Info
```

```
   0 ALERT level A = Most likely a serious problem - resolve or explain
   1 ALERT level B = A potentially serious problem, consider carefully
  30 ALERT level C = Check. Ensure it is not caused by an omission or oversight
  38 ALERT level G = General information/check it is not something unexpected

   0 ALERT type 1 CIF construction/syntax error, inconsistent or missing data
  34 ALERT type 2 Indicator that the structure model may be wrong or deficient
  17 ALERT type 3 Indicator that the structure quality may be low
  17 ALERT type 4 Improvement, methodology, query or suggestion
   1 ALERT type 5 Informative message, check
```

Validation response form

Please find below a validation response form (VRF) that can be filled in and pasted into your CIF.

```
# start Validation Reply Form
_vrf_PLAT084_lcz098
;
PROBLEM: High wR2 Value (i.e. > 0.25) ...................       0.27 Report
RESPONSE: ...
;
_vrf_PLAT088_lcz098
;
PROBLEM: Poor Data / Parameter Ratio ....................       9.26 Note
RESPONSE: ...
;
_vrf_PLAT213_lcz098
;
PROBLEM: Atom F00D            has ADP max/min Ratio .....        3.2 prolat
RESPONSE: ...
;
_vrf_PLAT218_lcz098
;
PROBLEM: Constrained U(i,j) Components(s) for C3                   2 Check
RESPONSE: ...
;
_vrf_PLAT234_lcz098
;
PROBLEM: Large Hirshfeld Difference N008     --C00C     .       0.16 Ang.
RESPONSE: ...
;
_vrf_PLAT241_lcz098
;
PROBLEM: High   'MainMol' Ueq as Compared to Neighbors of       C00C Check
RESPONSE: ...
;
_vrf_PLAT242_lcz098
;
PROBLEM: Low    'MainMol' Ueq as Compared to Neighbors of       C00B Check
RESPONSE: ...
;
_vrf_PLAT250_lcz098
;
```

```
PROBLEM: Large U3/U1 Ratio for <U(i,j)> Tensor(Resd    1)        2.1 Note
RESPONSE: ...
;
_vrf_PLAT260_lcz098
;
PROBLEM: Large Average Ueq of Residue Including        C1      0.103 Check
RESPONSE: ...
;
_vrf_PLAT334_lcz098
;
PROBLEM: Small <C-C> Benzene Dist.   C1       -C8       .       1.35 Ang.
RESPONSE: ...
;
_vrf_PLAT906_lcz098
;
PROBLEM: Large K Value in the Analysis of Variance ......      2.621 Check
RESPONSE: ...
;
_vrf_PLAT911_lcz098
;
PROBLEM: Missing FCF Refl Between Thmin & STh/L=    0.600         33 Report
RESPONSE: ...
;
_vrf_PLAT971_lcz098
;
PROBLEM: Check Calcd Resid. Dens.  1.00Ang From C5             2.30 eA-3
RESPONSE: ...
;
_vrf_PLAT972_lcz098
;
PROBLEM: Check Calcd Resid. Dens.  0.12Ang From C3            -2.04 eA-3
RESPONSE: ...
;
_vrf_PLAT973_lcz098
;
PROBLEM: Check Calcd Positive Resid. Density on      Ag01       1.06 eA-3
RESPONSE: ...
;
_vrf_PLAT976_lcz098
;
PROBLEM: Check Calcd Resid. Dens.  0.64Ang From N2      .      -0.96 eA-3
RESPONSE: ...
;
_vrf_PLAT977_lcz098
;
PROBLEM: Check Negative Difference Density on H12       .      -0.41 eA-3
RESPONSE: ...
;
# end Validation Reply Form
```

It is advisable to attempt to resolve as many as possible of the alerts in all categories. Often the minor alerts point to easily fixed oversights, errors and omissions in your CIF or refinement strategy, so attention to these fine details can be worthwhile. It is up to the individual to critically assess their own results and, if necessary, seek expert advice.

PLATON version of 17/09/2025; check.def file version of 17/09/2025

duplicate check

No duplication found

Datablock lcz098 - ellipsoid plot

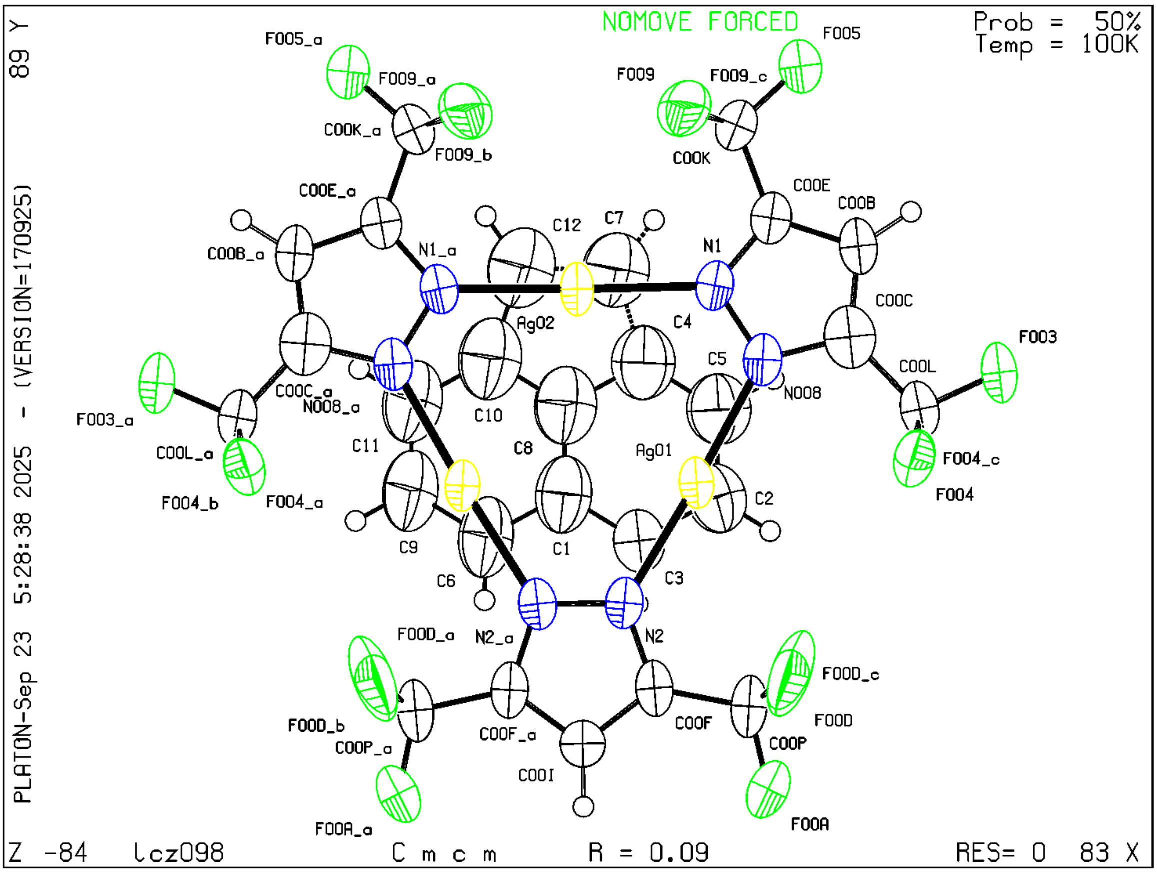

Supplement: Supplementary file 2 — Supporting File 2: anie72462‐sup‐0002‐DataFile.zip. [file ANIE-65-e1218503-s002.zip › supplementary_materials_crystallographic_data submit.zip!/cif_and_checkcif(1-114)/Ag3Pz3_98/Ag3Pz3_98_checkcif.pdf]
